# CARL – kontrollierte Reperfusion des ganzen Körpers: Von der kardiopulmonalen Reanimation zur zielgerichteten eCPR

**DOI:** 10.1007/s00398-022-00491-0

**Published:** 2022-02-18

**Authors:** C Benk, G Trummer, J.-S. Pooth, C. Scherer, F Beyersdorf

**Affiliations:** Klinik für Herz- und Gefäßchirurgie, Universitätsklinikum Freiburg, Hugstetter Str. 55, 79106 Freiburg, Deutschland

**Keywords:** Reanimation, Reperfusion, CPR, Ischämie‑/Reperfusionsschaden, Extrakorporale Zirkulation, Resuscitation, Reperfusion, CPR, Ischemia/reperfusion injury, Extracorporeal circulation

## Abstract

**Hintergrund:**

Inzidenz und Letalität des akuten Herz-Kreislauf-Stillstands sind seit Jahrzehnten gleichbleibend hoch.

**Fragestellung:**

Wie lassen sich die derzeit unbefriedigenden Ergebnisse nach einer Reanimation mit Blick auf das Überleben und die neurologischen, v. a. mit Blick auf die zerebralen Folgeschäden verbessern?

**Material und Methoden:**

Entwicklung eines therapeutischen Verfahrens zur Eindämmung des Ischämie‑/Reperfusionsschadens im Tiermodell. Entwicklung eines für die Reanimation optimierten Gerätesystems, mit dem sich eine kontrollierte Ganzkörperreperfusion auch außerklinisch umsetzen lässt.

**Ergebnisse:**

Etablierung der CARL-Therapie in der Klinik und in der Behandlung von OHCA-Patienten. Übernahme der Therapie und des CARL-Systems in eine klinische Beobachtungsstudie. Erste Fallberichte, in denen Patienten einen OHCA auch nach Ischämiezeiten bis zu 2 h ohne Schädigung des Gehirns überlebten.

**Schlussfolgerungen:**

Die CARL-Therapie eignet sich potenziell zur Behandlung reanimationspflichtiger Patienten mit einem auch über längere Zeit therapierefraktären Herz-Kreislauf-Stillstand.

## Abstract (en)

**Background:**

The incidence and mortality of acute cardiovascular arrest have been consistently high for decades.

**Objective:**

How to improve the currently unsatisfactory outcome after resuscitation regarding survival and neurological, especially cerebral, sequelae?

**Material and methods:**

Development of a therapeutic approach to curtail ischemia/reperfusion injury in an animal model. Development of a device system optimized for resuscitation that can be used to implement controlled reperfusion of the whole body outside the hospital.

**Results:**

Establishment of CARL treatment in the clinic and in the treatment of OHCA patients. Transfer of the CARL treatment and system in a clinical observational study. First case reports in which patients survived OHCA without brain damage even after ischemia times up to 2 h.

**Conclusion:**

CARL treatment is potentially suitable to treat patients suffering from cardiovascular arrest refractory to treatment even for prolonged periods.

Die CARL-Therapie (CARL: „*c*ontrolled *a*utomated *r*eperfusion of the who*l*e body“) ist ein Verfahren zur Behandlung von Patienten, die kardiopulmonal reanimiert werden müssen. Sie adressiert spezifisch die pathophysiologischen Stoffwechselvorgänge, die nach einem Herz-Kreislauf-Stillstand (HKS) unbehandelt zwangsläufig zum Tod bzw. im seltenen Überlebensfall häufig zu schweren neurologischen Schäden führen. Basis der neuen Methode ist eine zielgerichtete extrakorporale Reperfusion, in deren Verlauf die wichtigsten Vitalparameter des Patienten fortlaufend kontrolliert und angepasst werden. Der Organismus kann sich so zunächst schrittweise von der Mangeldurchblutung erholen und lässt sich anschließend so lange stabilisieren, bis die Ursache des HKS behoben ist.

## Krankheitsbild Herz-Kreislauf-Stillstand

Der akute HKS gehört nach wie vor zu den häufigsten Todesursachen weltweit. Verschiedene Krankheitsbilder können zu einem therapierefraktären Atem- und/oder Herz-Kreislauf-Versagen führen. In den meisten Fällen ist der Auslöser eine kardiale Grunderkrankung, z. B. eine koronare Herzkrankheit, die in ihrem schwersten Verlauf einen akuten Myokardinfarkt mit Herzstillstand auslösen kann.

In Europa erleiden rund 500.000 Menschen/Jahr einen plötzlichen Herzstillstand, den außerklinisch („out-of-hospital cardiac arrest“, OHCA) nur etwa 8 % und innerklinisch („in-hospital cardiac arrest“, IHCA) etwa 20 % aller Betroffenen überleben [1]. Etwa die Hälfte der Überlebenden zeigt nach der Reanimation dauerhaft gravierende neurologische Folgeschäden, die v. a. die Funktionalität des Zentralnervensystems betreffen [2, 3].

Der hohen Letalität stehen europaweit pro Jahr etwa 250.000 Reanimationsbehandlungen gegenüber. Die Ergebnisse dieser Behandlungen zu verbessern, ist eine der großen medizinischen Herausforderungen unserer Zeit.

## Herz-Lungen-Wiederbelebung

Die kardiopulmonale Wiederbelebung („cardiopulmonary resuscitation“, CPR) ist seit Jahrzehnten das Mittel der Wahl, um die Durchblutung des Myokards, des Gehirns und anderer Organe im Fall eines HKS aufrechtzuerhalten. Zu den Standardmethoden der CPR gehören – unabhängig von den Ursachen des kardiovaskulären Versagens – bis heute Herzdruckmassage, künstliche Beatmung, Defibrillation und die Injektion von Medikamenten.

Die Aussicht auf Erfolg im Rahmen einer CPR ist jedoch häufig gering, da sich u. a.

- die zugrunde liegende Ursache des HKS nicht vor Ort behandeln lässt und
- der Kreislauf trotz maximaler Bemühungen des Rettungsdienstes nicht spontan wieder in Gang kommt („return of spontaneous circulation“, ROSC).

In den letzten Jahren wurden deshalb umfangreiche Forschungsarbeiten durchgeführt und verbesserte Rettungsketten und intensive CPR-Trainingsprogramme implementiert. Auch neue therapeutische Ansätze wie die zielgerichtete CPR („targeted CPR“, tCPR) und die extrakorporale CPR (eCPR) sollten dazu beitragen, die Prognose für reanimierte Patienten zu verbessern [4–6].

### Targeted CPR

Die tCPR ist ein Konzept, für das spezifische hämodynamische, respiratorische und metabolische Ziele definiert wurden, die während der Reanimation erreicht werden sollen [4–6]. Ausschlaggebend sind hier u. a. die Kompressionstiefe der Herzdruckmassage, der arterielle Blutdruck, die endtidale CO_2_-Messung und die Titration des Sauerstoffs [4, 7]. Die Erweiterung des diagnostischen Spektrums war ein wichtiger Schritt hin zu einer rational begründeten und kontrollierbaren Therapie. Die Umsetzung der tCPR in die Praxis erweist sich allerdings als schwierig, da außerklinisch kaum geeignete Überwachungsmaßnahmen verfügbar sind. Entsprechend limitiert sind auch die therapeutischen Optionen.

### Extrakorporale CPR

Ähnliche Einschränkungen gelten für die Anwendung extrakorporaler Kreislaufunterstützungssysteme (auch bekannt als: „extracorporeal life support“, ECLS), die zunehmend zur Reanimation eingesetzt werden. Obwohl der Blutkreislauf und die Atemfunktion mit deren Hilfe schnell ersetzt werden können, mangelt es auch in diesem Umfeld an einem schnell verfügbaren Monitoring. Deshalb ist eine zielgerichtete Behandlung reanimationspflichtiger Patienten auch mit der eCPR nur sehr eingeschränkt möglich [8].

Trotzdem hat das Konzept der eCPR die Vision gefördert, dass sich die Prognose für reanimierte Patienten tatsächlich verbessern lässt. Deshalb wurde das Verfahren 2015 in Form spezifischer CPR-Algorithmen bzw. als „Alternative zur konventionellen CPR bei ausgewählten Patienten“ in die Leitlinien integriert [9, 10].

Erste Studienergebnisse deuten darauf hin, dass sich die Erfolgsbilanz nach Reanimation mit Blick auf das Überleben tatsächlich verbessert [11]. Hinsichtlich der neurologischen Erholung erfordert der Status quo dennoch neue Ansätze und Ideen, um das therapeutische Potenzial der eCPR voll auszuschöpfen.

## Ischämie‑/Reperfusionsschaden

Als wesentliche Ursache des schlechten Outcome nach einem HKS ist seit Jahrzehnten der Ischämie‑/Reperfusionsschaden (IRI) Gegenstand der Forschung. Nach heutigem Erkenntnisstand verursacht die abrupte Unterbrechung des Blutflusses (Ischämie) zeitabhängig eine zelluläre Schädigung, die hauptsächlich auf einen Substratmangel zurückzuführen ist [12–14].

### Ischämie

Der ischämisch bedingte Sauerstoffmangel führt zu einer Dysfunktion der zellulären Ionentransporter und damit zu einer eingeschränkten Osmoregulation. Die Depolarisation des Membranpotenzials bewirkt einen Kaliumausstrom, während Kalzium‑, Chlorid- und Natriumionen unkontrolliert in die Zellen einströmen, wodurch verstärkt Wasser eingelagert wird. Die Ödembildung wirkt sich im Endothelgewebe der Arterien besonders fatal aus, v. a. im Gehirn, wo die Einengung des Gefäßlumens kleinerer Gefäße (z. B. der Arteriolen) zu einer weiteren Einschränkung oder zum Stillstand der Blutzirkulation führt. Abbauprodukte sammeln sich an, der zelluläre Stoffwechsel gerät zunehmend außer Kontrolle, und wichtige Zellstrukturen werden zerstört. Dauert die Ischämie länger an, sterben die Zellen ab, was letztlich nicht nur zum Tod eines Organs, sondern des ganzen Organismus führt. Insgesamt äußert sich eine ausgeprägte Ischämie in der folgenden Symptomatik:

- Energieverlust (nahezu vollständiger Abbau von ATP binnen weniger Minuten),
- metabolische Acidose,
- Zunahme des intrazellulären Wassergehalts,
- Vasoplegie mit anschließender Vasodilatation und Hypotension.

### Reperfusion

**Figure 1:**
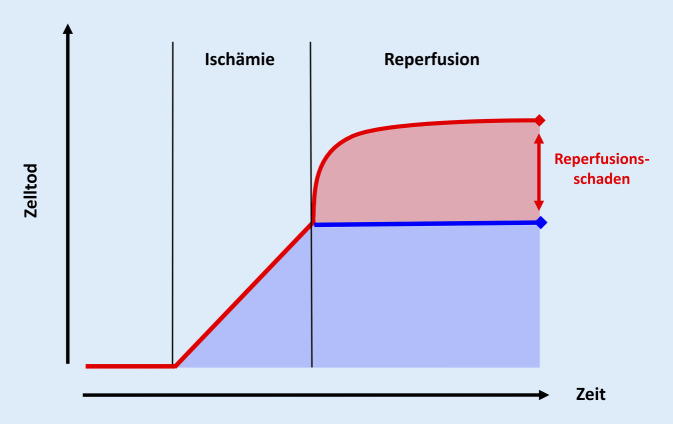

Ziel der derzeit gängigen Methoden der CPR ist es, den Organismus möglichst schnell wieder mit Nährstoffen zu versorgen, sodass der Zellstoffwechsel wieder in Gang kommt. Die ischämische Zelle ist jedoch gegenüber weiteren Schädigungen extrem empfindlich, und so ist es häufig gerade die Reperfusion infolge einer Reanimation, die das Absterben der Zellen noch beschleunigt. Denn eine (unkontrollierte) Reperfusion kann in den vorgeschädigten Zellen eine Reihe von Stoffwechselprozessen triggern, die selbst schwere Zellschäden bis hin zum Zelltod verursachen. Das Ausmaß der zellulären Schädigung nimmt deshalb in der Phase der Reperfusion zunächst noch zu (Abb. 1).

Eine wichtige Rolle spielt in dieser Reaktionskette der Sauerstoff. Um die Blutzirkulation und die Versorgung des Gewebes wieder in Gang zu setzen, wird dem Betroffenen im Falle eines akuten Herz-Kreislauf-Versagens u. a. meist auch reiner Sauerstoff zugeführt. Dadurch wird im ohnehin geschwächten Organismus die Entstehung von Sauerstoffradikalen begünstigt, die ihrerseits eine Vielzahl an Zellschäden auslösen können.

Der Reperfusionsschaden tritt innerhalb von Sekunden oder Minuten nach der Wiederherstellung des Blutflusses zum ischämischen Gewebe auf. Gegenmaßnahmen müssen entsprechend zeitnah implementiert werden.

## Targeted eCPR – die CARL-Therapie

Die ischämisch induzierten pathophysiologischen Stoffwechselanpassungen nach einem akuten Herzstillstand lassen sich nicht verhindern. Es ist jedoch möglich, Gewebe und Organe auch nach längeren Ischämiezeiten zu schützen und v. a. den Reperfusionsschaden einzudämmen, wenn die Reperfusion kontrolliert durchgeführt wird [14]. Das zeigen die heute obligaten therapeutischen Verfahren in der Herzchirurgie, der Organtransplantation sowie bei der Reperfusion von Extremitäten [16–19]. Kernelemente dieser Verfahren sind:

- die Kontrolle der physikalischen Reperfusionsbedingungen (Blutdruck, Blutfluss, Pulsatilität, Bluttemperatur),
- die situative Modifikation der Reperfusionslösung, in der Regel des rezirkulierenden Blutes, durch Anpassung des Sauerstoff- und Kohlendioxidgehalts, des pH-Werts, des Elektrolytgehalts und der Osmolarität,
- ein schnell verfügbares, umfassendes Monitoring.

Anhand dieser Vorgaben und auf der Basis einer intensiven Forschungsarbeit [20–26] wurde in den letzten 16 Jahren das Konzept der kontrollierten Ganzkörperreperfusion („*c*ontrolled *a*utomated *r*eperfusion of the who*l*e body“, CARL) entwickelt. In deren Mittelpunkt steht die Vorbereitung des durch den Sauerstoffmangel geschädigten Gewebes auf das Wiedereinsetzen des körpereigenen Blutkreislaufs. Der Patient wird dazu über die Leistengefäße an eine extrakorporale Zirkulation angeschlossen, anschließend wird das venöse Blut mithilfe eines Oxygenators spezifisch mit Sauerstoff angereichert, patientenindividuell modifiziert und dann in den Körper zurückgepumpt.

### Kontrolle der physikalischen Reperfusionsbedingungen

Zu den physikalischen Parametern, über die sich ein Ischämie‑/Reperfusionsschaden eindämmen lässt, gehören neben dem Reperfusionsdruck der korrespondierende Reperfusionsfluss und die Körpertemperatur. Diese 3 Größen werden im Rahmen der CARL-Therapie, wie in Tab. 1 beschrieben, situativ spezifisch reguliert.

**Table 1:** 

Parameter/Zielwert	Beschreibung/Rationale
Pulsatilität	Die Pulsation erhöht die hämodynamische Kraft zur Wiederöffnung von Kapillarstromgebieten
Blutfluss und Blutdruck	Der Perfusionsdruck während einer CARL-Therapie korrespondiert mit einem Perfusionsfluss (Pumpenminutenvolumen) und einem Blutfluss, der annähernd den Verhältnissen in einem gesunden Organismus entspricht
Hypothermie (Venöse Bluttemperatur)	Die Organschädigung nach Herz-Kreislauf-Stillstand lässt sich auch über ein Abkühlen des Körpers eindämmen, denn Kälte verlangsamt den Zellstoffwechsel, drosselt den Sauerstoffbedarf und senkt den zellulären Energieverbrauch

### Patientenindividuelle Anpassung des Reperfusats

Die Zusammensetzung der CARL-Reperfusionslösung wird mithilfe spezifisch wirksamer Substanzen an den Bedarf und die Pathophysiologie des ischämischen Gewebes angepasst. Das perfundierende Blut wird dazu, wie in Tab. 2 beschrieben, modifiziert.

**Table 2:** 

Parameter/Zielwert	Beschreibung/Rationale
Sauerstoff (p_a_O_2_)	Das Patientenblut wird mit einem Luftgemisch aus Atemluft und Sauerstoff (Sauerstoffgehalt 21–100 %) angereichert, um die Bildung von Sauerstoffradikalen zu limitieren (normoxische Reperfusionsstrategie)
Kohlendioxid (p_a_CO_2_)	Die CARL-Therapie setzt initial auf eine vorsichtige, über den CO_2_-Gehalt gesteuerte Blutpufferung (pH-stat-Strategie), da der sofortige Ausgleich der Übersäuerung (z. B. durch Puffer) den ohnehin gestörten Zellstoffwechsel weiter überlasten würde. Im Verlauf einer milden Acidose können die Zellen dagegen schrittweise Substrate aufnehmen (reduzierte metabolische Aktivität in der Phase des Substratmangels) und Voraussetzungen für Reparaturprozesse schaffen
Osmolarität im Blutserum	Das Blut wird in der ersten Phase der CARL-Therapie mithilfe osmoaktiver Substanzen (Natrium, Humanalbumin, Mannitol) auf einen hyperosmolaren Wert gebracht. Dieser Ansatz wirkt der Bildung von Hirnödemen entgegen, indem er die Wasserbindekraft des Blutes erhöht. Dem Zellgewebe wird infolgedessen Wasser entzogen, was das Abschwellen der Gefäßendothelien begünstigt
Kalium	Um den Sinusrhythmus wiederherzustellen und den Sauerstoffverbrauch im Myokard zu minimieren, wird das Herz ggf. vorübergehend mithilfe einer Kaliumlösung pharmakologisch stillgestellt (sekundäre Kardioplegie)
Kalzium (ionisiertes Kalzium)	Der unkontrollierte Kalziumeinstrom in die ischämische Zelle befördert die Bildung von Hirnödemen, deshalb wird der Kalziumgehalt zu Beginn der CARL-Therapie mithilfe von Zitrat abgesenkt
Natrium	Der ischämie-/reperfusionsbedingt unphysiologische Gehalt an Natriumionen in der Zelle und im Gewebe begünstigt die Ausbildung von Ödemen. Zur Eindämmung des Ischämie‑/Reperfusionsschadens werden deshalb Natriumionen in Form von Kochsalzlösung zugeführt
Arterieller pH-Wert	Die pH-stat-Strategie dient der Reduktion der metabolischen Aktivität während des Substratdefizits

Zur Wiederherstellung des Säure-Basen-Gleichgewichts wird außerdem fortlaufend der Base Excess überwacht. Über den Hämatokrit bzw. die Hämoglobinkonzentration werden die Sauerstoffbindungskapazität und die Viskosität des Blutes angepasst. Letzteres senkt das Risiko einer Thrombenbildung währen der extrakorporalen Zirkulation.

### Medikation

Die kontrollierte Ganzkörperfusion wird im Rahmen einer CARL-Therapie auch medikamentös unterstützt. So wird das Blut eines mit CARL behandelten Patienten unmittelbar mit Beginn der Therapie heparinisiert, zusätzlich unterstützt das im Priming enthaltene Zitrat die sofortige Antikoagulation. Eine Antikoagulation ist angezeigt, weil über den Kontakt des Blutes mit den künstlichen Oberflächen der EKZ das Gerinnungs- und Immunsystem aktiviert wird. Die Gerinnungsreaktion setzt in diesem Fall schlagartig ein (Hyperkoagulation); schwerwiegende pulmonale, renale, neurologische und hämodynamische Komplikationen bis hin zu Organdysfunktion und Organversagen können die Folge sein. Zur Überwachung der Antikoagulation und zur Verhinderung von fatalen Komplikationen werden deshalb die folgenden Parameter fortlaufend kontrolliert: aPTT, ACT, Heparinkonzentration, Anti-Faktor-Xa-Aktivität.

Um die Antikoagulation anzupassen und systemimmanente Thrombenbildungen zu verhindern, wird ggf. auch das Pumpenminutenvolumen angepasst.

Als Antiarrhythmika und zur Neuroprotektion werden dem Reperfusat Magnesium und Lidocain zugesetzt. Das Magnesium dient dem zusätzlichen Schutz der Mitochondrien.

Inotrope Substanzen wie Suprarenin o. Ä., die die Kontraktionskraft des Herzens beeinflussen, werden in der Frühphase der CARL-Therapie (innerhalb der ersten 30 min) möglichst nicht eingesetzt.

### Fortlaufendes Monitoring

Die kontinuierliche Überwachung aller behandlungsrelevanten Parameter und die Möglichkeit, diese adäquat und schnell anzupassen, sind essenzielle Voraussetzungen für die erfolgreiche Versorgung akut und kritisch kranker Patienten. Daher wurden für die CARL-Therapie der Umfang und die Anforderungen an ein geeignetes Monitoring und an eine patientenindividuelle Steuerung der physikalischen und biochemischen Reperfusionsbedingungen anhand der Funktionalitäten einer Herz-Lunge-Maschine neu definiert. Folgerichtig wurden u. a. eine kontinuierliche venöse und arterielle Blutgasanalyse (BGA) und eine arterielle Blutdrucküberwachung in das CARL-Setting implementiert.

## Das CARL-System

**Figure 2:**
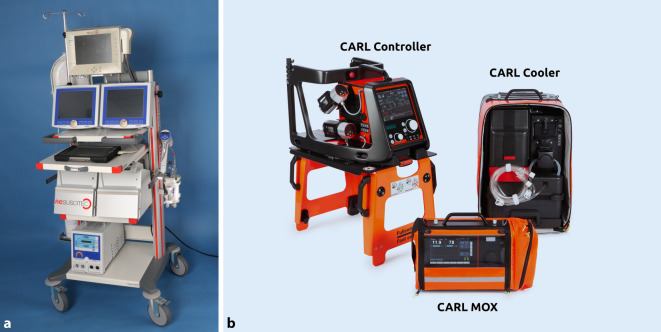

Um die CARL-Therapie umsetzen zu können, musste ein neues Gerätesystem entwickelt werden, dessen funktionales Spektrum sowohl ein umfangreiches und schnell verfügbares Monitoring als auch eine präzise Steuerung der physikalischen und biochemischen Reperfusionsbedingungen ermöglicht. Da ein solches Gerät bisher nicht verfügbar war, wurde zur ersten Anwendung von CARL bei Patienten mit akutem HKS und anschließender verlängerter CPR die Systemkonfiguration CIRD 1.0 (Controlled Integrated Resuscitation Device 1.0; Abb. 2) entwickelt und zugelassen. Diese Konfiguration, die nur für den innerklinischen Einsatz vorgesehen war, basierte auf einer erweiterten ECLS, die u. a. ein Online-Blutgas-Monitoring, eine kontrollierte Sauerstoffzufuhr, das Anlegen eines hohen und pulsierenden Blutflusses und eine entsprechende Kühlvorrichtung zur Induktion einer Hypothermie umfasste.

Die Systemfügung CIRD 1.0 war der Vorläufer des heutigen, CE-zertifizierten CARL-Systems (Resuscitec GmbH, Freiburg; Abb. 2), dessen tragbare Hauptkomponenten sowohl für den innerklinischen als auch speziell für den außerklinischen Einsatz entwickelt wurden.

Das System besteht aus einer zentralen Kontroll- und Steuereinheit (CARL Controller), an die der Patient über die Leistengefäße angeschlossen wird. Dazu kommen ein Sauerstoffkontrollgerät (CARL MOX) und eine Versorgungseinheit zur Applikation einer therapeutischen Hypothermie (CARL Cooler). Die 3 Hauptkomponenten des CARL-Systems entsprechen den Anforderungen der CARL-Therapie im Umfeld einer OHCA u. a. durch die folgenden Leistungsmerkmale:

- mobiles Doppelpumpenkontrollsystem zur Erzeugung eines hohen, pulsatilen Blutflusses,
- arterielle Blutgasanalyse,
- Plug-in-Fiberoptikkatheter zur Messung des intraarteriellen Blutdrucks,
- schneller Systemstart mit einem vorgefertigten Perfusionsset mit integriertem Druck‑, S_v_O_2_- und Temperatursensor,
- spezielle Priming-Lösung,
- mobiles Sauerstoffkontrollgerät mit Blower-Technologie,
- mobiles Hypothermiegerät zum schnellen Abkühlen des Patienten,
- Transportausrüstung für alle Komponenten.

Die erweiterten diagnostischen und therapeutischen Möglichkeiten der CARL-Systematik sind im Vergleich zu den derzeit gängigen Methoden der kardiopulmonalen Wiederbelebung in Tab. 3 zusammengefasst.

**Table 3:** 

Option	CARL	eCPR	CPR
Venoarterielle Perfusion und Oxygenierung	✔	✔	(✔)
Hoher arterieller Perfusionsdruck	✔	–	–
Pulsatiler arterieller Blutfluss	✔	–	–
Hoher arterieller Blutfluss	✔	–	–
Sofortige Hypothermie	✔	–	–
Kontinuierliche BGA (venös und arteriell)	✔	–	–
Kontrollierte Oxygenierung	✔	–	–
Hypokalzämie	✔	–	–
Hyperkaliämie	✔	–	–
Hyperosmolarität	✔	–	–

## Studienlage

### Präklinik

Das CARL-Gesamtkonzept und seine einzelnen Komponenten wurden zunächst in zahlreichen chronischen Tierversuchen getestet und weiterentwickelt [20–27]. Das entsprechende Großtiermodell wurde etabliert, um die Rahmenbedingungen für eine erfolgreiche Reperfusion nach schwerer Ischämie mit Blick auf die Endpunkte Letalität und neurologische Erholung zu untersuchen [23]. Die Tiere wurden dafür für 15 min bzw. 20 min ungeschützt und ohne jeden Versuch der Wiederbelebung einer warmen Ischämie ausgesetzt [20, 23, 24]. In der anschließenden Reperfusionsperiode von 60 min wurden die Reperfusionsbedingungen und das zirkulierende Blut entsprechend den im Rahmen einer Tieroperation kontinuierlich verfügbaren Messparametern adaptiert. Je nach untersuchter Variable überlebten bis zu 90 % der Tiere den Versuchsverlauf, davon zeigten wiederum 90 % eine vollständige neurologische Erholung [20, 23–26]. Anhand der Zusammenschau aller bekannten Publikationen zur Reperfusion einzelner Organe sowie der Ergebnisse der beschriebenen Tierversuche war es möglich, für die CARL-Therapie eine Reihe von zielführenden Behandlungseckpunkten zu definieren.

### Anwendungsbeobachtung

Die beschriebenen Elemente einer kontrollierten Reperfusion des ganzen Körpers wurden am Universitätsklinikum Freiburg in einer krankenhausbasierten Standardarbeitsanweisung (SOP) zusammengefasst. Dieses Dokument bietet in der hochgradig interaktiven Situation einer fortgesetzten CPR, in der ein Bedarf an extrakorporaler Unterstützung auftritt, eine Anleitung für alle Mitglieder des behandelnden Teams.

Auf dieser Grundlage wurden am Universitätsklinikum Freiburg im Rahmen einer Anwendungsbeobachtung in einer unkontrollierten, konsekutiven Serie *n* = 14 Patienten mit CARL behandelt (DRKS00005773) [28]. In allen Fällen ereignete sich der HKS außerhalb einer Klinik im Beisein von Zeugen, und die CPR wurde sofort eingeleitet. Die interdisziplinären Teams entschieden sich jeweils erst nach einer verlängerten CPR bis zu 2 h für die Anwendung der CARL-Therapie. Alle Patienten wurden in der Folge gut überwacht und gemäß den genannten Vorgaben behandelt. Die Ergebnisse dieser Behandlungsserie stimmten mit den Erkenntnissen aus den präklinischen Experimenten überein: Trotz der z. T. extrem langen vorangegangenen CPR-Periode (zwischen 51 und 120 min) überlebten 7/14 Patienten und erlangten das volle Bewusstsein wieder. Dabei konnten 6/7 der Cerebral Performance Class (CPC) „1“ zugeordnet werden. Eine vollständige zerebrale Erholung zeigte auch die Patientin mit der längsten Vorlaufzeit (120 min CPR), die trotz einer verbleibenden Parese der Beine aufgrund eines A.-spinalis-anterior-Syndroms heute wieder in ihrem Beruf arbeitet [29].

### PMCF-Studie

Das CARL-System wird derzeit in einer „Post-market-clinical-follow-up“-Studie (PMCF-Studie) in mehreren deutschen und europäischen Kliniken getestet (DRKS00018967). Die Rekrutierung für diese Studie wurde jedoch durch die SARS-CoV-2-Pandemie verzögert und wird voraussichtlich in 12 bis 18 Monaten abgeschlossen sein.

## Fazit für die Praxis

- Die Ergebnisse im Bereich der Reanimation sind seit Jahrzehnten gleichbleibend unbefriedigend.
- Europaweit überleben nur rund 8 % einen OHCA.
- Der seltene Überlebensfall geht häufig mit schweren neurologischen Schäden, v. a. im Zentralnervensystem, einher.
- Die CARL-Therapie adressiert den Ischämie‑/Reperfusionsschaden, der nach neueren Erkenntnissen für die schlechten Ergebnisse nach Reanimation mitverantwortlich ist.
- Kernelemente des neuen Verfahrens sind die kontinuierliche Überwachung und patientenindividuelle Modifikation relevanter Parameter, eine kontrollierte Sauerstoffzufuhr und die Induktion einer therapeutischen Hypothermie.
